# ^1^H NMR metabolic profiling of cod (*Gadus morhua*) larvae: potential effects of temperature and diet composition during early developmental stages

**DOI:** 10.1242/bio.014431

**Published:** 2015-11-06

**Authors:** Matilde Skogen Chauton, Trina Falck Galloway, Elin Kjørsvik, Trond Røvik Størseth, Velmurugu Puvanendran, Terje van der Meeren, Ørjan Karlsen, Ivar Rønnestad, Kristin Hamre

**Affiliations:** 1 Department of Marine Resources Technology, SINTEF Fiskeri og Havbruk, Trondheim N-7465, Norway; 2 Department of Biology, Norwegian University of Science and Technology (NTNU), Trondheim N-7491, Norway; 3 Department Environmental Technology, SINTEF Materialer og kjemi, Trondheim N-7465, Norway; 4 Nofima Marine, PO Box 6122, Tromsø N-9291, Norway; 5 Institute of Marine Research, Austevoll Research Station, and Hjort Centre for Marine Ecosystem Dynamics, Storebø NO-5392, Norway; 6 Department of Biology, University of Bergen, Bergen N-5020, Norway; 7 National Institute of Nutrition and Seafood Research (NIFES), PO Box 2029, Bergen N-5817, Norway

**Keywords:** *Gadus morhua*, Larvae development, Temperature, Nutrition, metabolomics

## Abstract

Marine aquaculture offers a great source of protein for the increasing human population, and farming of, for example, Atlantic salmon is a global industry. Atlantic cod farming however, is an example of a promising industry where the potential is not yet realized. Research has revealed that a major bottleneck to successful farming of cod is poor quality of the larvae and juveniles. A large research program was designed to increase our understanding of how environmental factors such as temperature and nutrition affects cod larvae development. Data on larvae growth and development were used together with nuclear magnetic resonance. The NMR data indicated that the temperature influenced the metabolome of the larvae; differences were related to osmolytes such as betaine/TMAO, the amino acid taurine, and creatine and lactate which reflect muscle activity. The larvae were fed *Artemia* from stage 2, and this was probably reflected in a high taurine content of older larvae. Larvae fed with copepods in the nutrition experiment also displayed a high taurine content, together with higher creatine and betaine/TMAO content. Data on the cod larvae metabolome should be coupled to data on gene expression, in order to identify events which are regulated on the genetic level versus regulation resulting from temperature or nutrition during development, to fully understand how the environment affects larval development.

## Abstract (precis)

**Summary:** Metabolomic ‘snapshots’ from developing cod larvae reflect the temperature and diet experienced from hatching to juvenile and provide insight into how important processes such as osmoregulation and muscle development might be affected.

## INTRODUCTION

Seafood is one of the major protein sources for human consumption in the world today and the importance of seafood proteins is likely to increase in the coming years. Marine aquaculture has become a knowledge based industry that successfully supplies a large fraction of high quality food. Farming of Atlantic salmon (*Salmo salar* Linnaeus) is one example of a successful aquaculture industry, and cod (*Gadus morhua* L.) farming has been attempted for some decades but with less success compared to salmon and other species. One of the main bottlenecks of successful cod farming is the poor larval and juvenile quality (Hamre et al., 2013b; Valente et al., 2013).

Teleosts are ectothermic and the early development is therefore closely correlated to water temperature. Lower temperatures result in slower development, later hatching and larvae with a smaller yolk sac at hatching (Galloway et al., 1998; Geffen et al., 2006; Hall et al., 2003; Hall and Johnston, 2003; Hunt von Herbing et al., 1996; Jobling, 2002; Johnston et al., 2009). The optimal temperature for embryonic development in cod is still being debated (Puvanendran et al., 2013), and although it has been shown that high temperatures result in rapid growth and large larvae at hatching, harmful effects may appear at later stages. Development and metabolic responses at different temperature regimes need to be examined in order to increase our understanding of the effects of temperature on larval growth. Such knowledge will ensure production of high quality cod larvae in the hatcheries.

Nutrition is important from the moment the cod larvae start to feed, and food quality determines the development of the young. In the sea and in semi-natural pond systems, cod larvae feed on zooplankton and especially copepod nauplii (Last, 1978; van der Meeren and Næss, 1993). Large scale production of copepods for use in hatcheries is challenging, and rotifers and *Artemia* Leach (which are relatively easy to cultivate) have therefore been used to feed fish larvae in commercial production. It has become clear; however, that rotifers and *Artemia* are not nutritionally optimal for the larval cod development (Busch et al., 2010; Hamre et al., 2008; Li et al., 2015; Maehre et al., 2013; van der Meeren et al., 2008), and cod larvae fed with zooplankton or nauplii from *Acartia tonsa* Dana showed higher growth rate and less bone deformities compared to larvae fed with rotifers (Busch et al., 2010; Finn et al., 2002; Imsland et al., 2006; L. R. McQueen, PhD thesis, University of Tromsø, 2003; Otterlei et al., 1999; Øie et al., 2015). Copepods have a well-balanced composition of proteins, free amino acids and lipids, including significant amounts of n-3 fatty acids in the phospholipids (Drillet et al., 2006; Evjemo et al., 2003; Olsen et al., 2014; Shields et al., 1999; van der Meeren et al., 2008). Changing diets from rotifers to copepods have shown promising results in several aquatic species (Conceicao et al., 2010).

The underlying mechanisms that result in differences in growth and quality of fish larvae is still unknown, but one way to learn more about this is to study the metabolome of developing larvae. The metabolome is the set of small molecules (<1500 D) present in a cell, and metabolomics is the study of interactions between the environment and the metabolome of an organism (or parts of it). We have used proton nuclear magnetic resonance (NMR) spectroscopy to analyze tissue extracts and identify metabolites that can be used to follow environmental impacts on the fish larvae. ^1^H NMR has been used to study the metabolic profile in gilthead sea bream *Sparus aurata* L. (Picone et al., 2011; Savorani et al., 2010), effects of larval feeding in ballan wrasse *Labrus bergylta* Ascanius (Øie et al., 2015), and effects of feeding (Bankefors et al., 2011) or stress response (Karakach et al., 2009) on Atlantic salmon. NMR has also been used to study cod in food processing contexts, often using ^31^P NMR (Sartoris et al., 2003) or ^13^C NMR (Standal et al., 2008); ^1^H NMR on the other hand, has been used to study lipid hydrolysis and esterification in cod gonads (Falch et al., 2007) or bioactive compounds in cod fillet (Martinez et al., 2005). There are studies which have used ^1^H NMR to examine the environmental effects such as temperature (Turner et al., 2007) or toxic substances (Viant et al., 2006) on the metabolome of developing fish larvae. There are, however, few studies that have applied a metabolomics approach employing ^1^H NMR to study environmental effects such as temperature or diet on the early development of cod larvae.

The present study is part of a larger program where the aim is to build a knowledge platform to understand the environmental and nutritional impacts on the early development, growth and metabolism in Atlantic cod larvae. The present study uses ^1^H NMR spectroscopy analyses and unsupervised cluster analyses on data from two separate experiments targeting effects of (1) temperature and (2) diet composition on the metabolism and growth of early stages of Atlantic cod larvae. The main objective of the knowledge platform was to understand the reasons for high larval mortality or poor physiological conditions of the survivors and also long-term effects that appear later in life as a result of the environmental and nutritional influences in early life.

## RESULTS

A list of all the identified compounds in the ^1^H NMR spectra from cod larvae is given in Table 1. Our NMR data showed the presence of metabolites such as 14 different amino acids, organic acids/osmolytes betaine, choline and lactate, N,N-Dimethylglycine and taurine (Tau). We also identified TMAO, the tricarboxylic acid cycle intermediate succinate, as well as formate and 4-Aminonbutyrate, energy compounds glucose, creatine and ATP, in addition to some fatty acid metabolism intermediates.

**Table 1.**
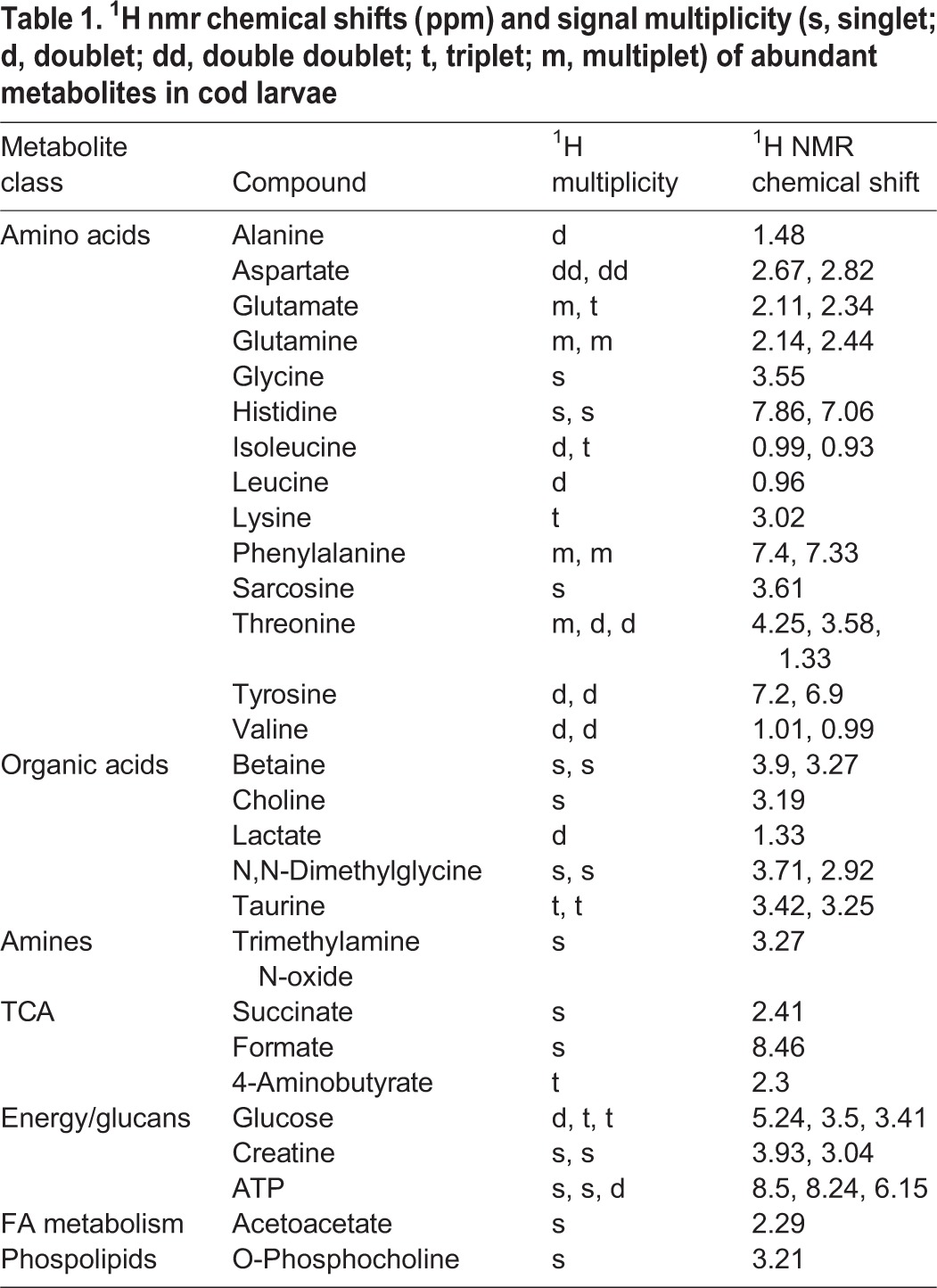
**^1^H nmr chemical shifts (ppm) and signal multiplicity (s, singlet; d, doublet; dd, double doublet; t, triplet; m, multiplet) of abundant metabolites in cod larvae**

The experimental setup with differences in temperature and diets resulted in different developmental times for the larvae, and therefore different sizes at the sampling times. A staging system based on larvae from both the temperature and the nutrition experiments was established and used here, see Materials and Methods for a description of cod stages (Table 2), and the length and stages of cod larvae at each sampling (Table 3).

**Table 2.**
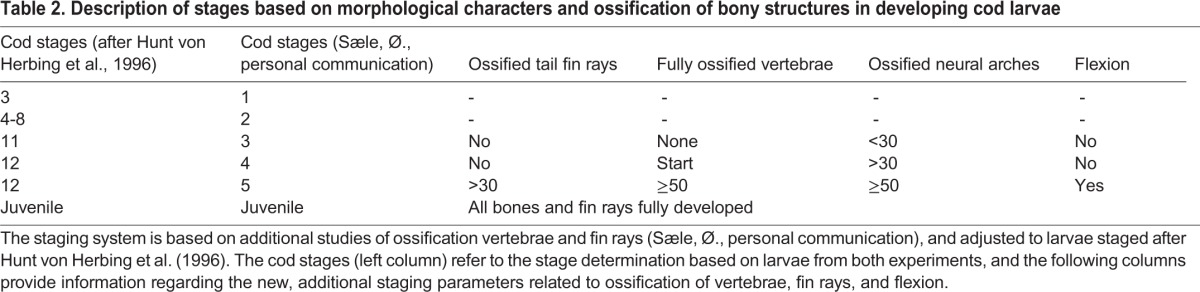
**Description of stages based on morphological characters and ossification of bony structures in developing cod larvae**

**Table 3.**
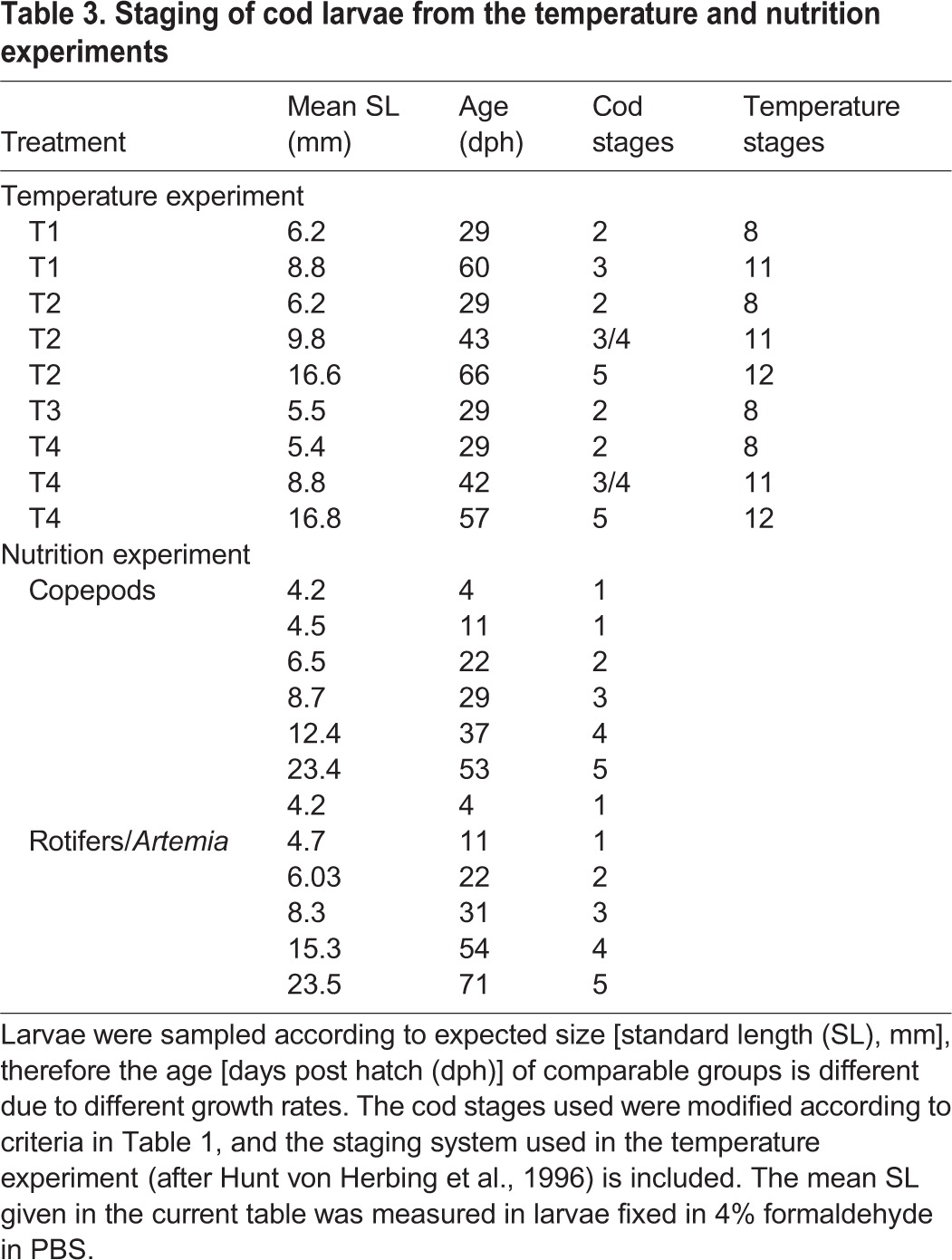
**Staging of cod larvae from the temperature and nutrition experiments**

### Temperature experiment

In the temperature experiment, cod larvae stages and average standard lengths was (stage/length, in mm): 2/6.2, 3/8.8 in T1 treatment, 2/6.2, 3-4/9.8, and 5/16.6 in T2. In the T3 treatment larvae reached 5.5 mm at stage 2, while in the T4 treatment larvae at stage 2 were on average 5.4 mm long, in stage 3-4 they were 8.8 and at stage 5 they were 16.8 mm long (Table 3). There were no significant differences in larval growth in the four treatments during the first 20-25 days (stage 1-2), but after that the larvae reared at the highest temperature (T2 and T4) grew faster than the other larval groups (Puvanendran et al., 2013). The T3 larvae were only measured during the first phase of the experiment, and the measurements indicated that they grew with the same speed as the T1 larvae. Due to differences in growth rates and development between the treatments the larvae reached different stages on the selected sampling days (Table 3). In the control treatment (T1), stage 2 and 3 corresponded to 29 and 60 days post hatch (dph), respectively. In the T2 treatment stage 5 corresponded to 66 dph, while in the T4 treatment stage 5 corresponded to 57 dph. Of the larvae ranging from 8.7 to 9.8 mm standard length (SL), the T1 larvae were less developed compared to larvae from the T2 and T4 treatments (Table 2). Only larvae from T2 and T4 were sampled for staging after stage 3/4.

**Fig. 1.**
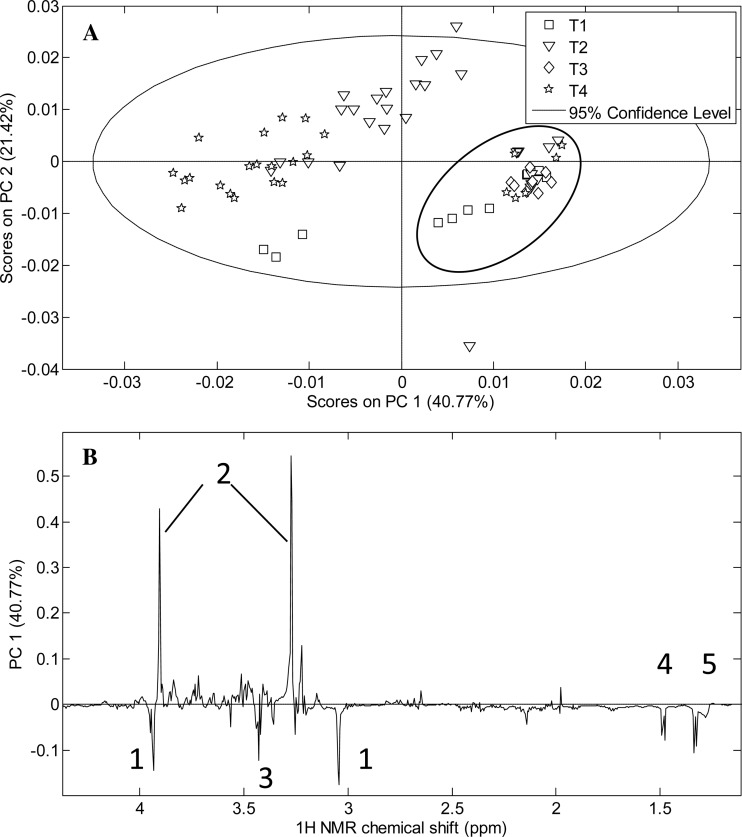
**Principal component analysis (PCA) score plot of larvae from developmental stages 1-5 in the temperature experiment.** (A) Symbols refer to the different temperature regimes: T1 (square), low temperature during all phases; T2 (triangle), low temperature during egg phase and high temperature during larva and juvenile phase; T3 (diamond), high temperature during egg phase, low temperature during larva and juvenile phase; T4 (star), high temperature during egg and larva phase, low temperature during juvenile phase. Thin line is the 95% C.I. of the PCA, and the bold black circle has been drawn by hand to identify the grouping of individuals of stage 2 to the right (independent of temperature regime) versus the individuals of stages 3 to 5 which show a tendency of grouping but with larger individual variance (to the left). (B) Loading plot of the first principal component (PC1) in the PCA analysis. Numbered peaks show (1) creatine, (2) betaine/TMAO, (3) taurine, (4) alanine and (5) lactate.

The NMR data from the temperature experiment were analysed by PCA, and there was a clear distinction between younger larvae (stage 2) and the older larvae (stages 3-5). Because we pooled several larvae from the early samplings they cluster tightly, while the score plot shows some inter-individual variance in the older larvae that were analyzed one by one from the T2 and T4 treatments (Fig. 1A). The PC1 loading plot indicated increasing amounts of N, N, N-trimethylamine (betaine)/trimethylamine N-oxide (TMAO) and less lactate, alanine (Ala), Tau, and creatine (Fig. 1B). The scores along PC2 (data not shown) indicated that the betaine/TMAO, Ala and lactate levels were higher in the T2 larvae than in the T4 larvae.

### Nutrition experiment

In the nutrition experiment the larvae sizes at the different stages were as follows (stage/length, in mm): 1/4.2-4.5, 2/6.5, 3/8.7, 4/12.4 and 5/23.4 in the copepod regime, and 1/4.2-4.7, 2/6.03, 3/8.3, 4/15.3 and 5/23.5 in the rotifer/*Artemia* regime (Table 3). Larvae in the nutritional experiment grew at similar rates until 22 dph (stage 2). Thereafter and until stage 4, the daily length growth was 4.4% in larvae fed copepods, while in larvae fed rotifers/*Artemia* the daily length growth was only 2.2%. Larvae weaned onto a formulated diet after stage 4 again grew at similar rates (Karlsen et al., 2015). Samples were taken at comparable larval sizes, and the stages corresponded well with the larval sizes for both feeding regimes (Table 3).

**Fig. 2.**
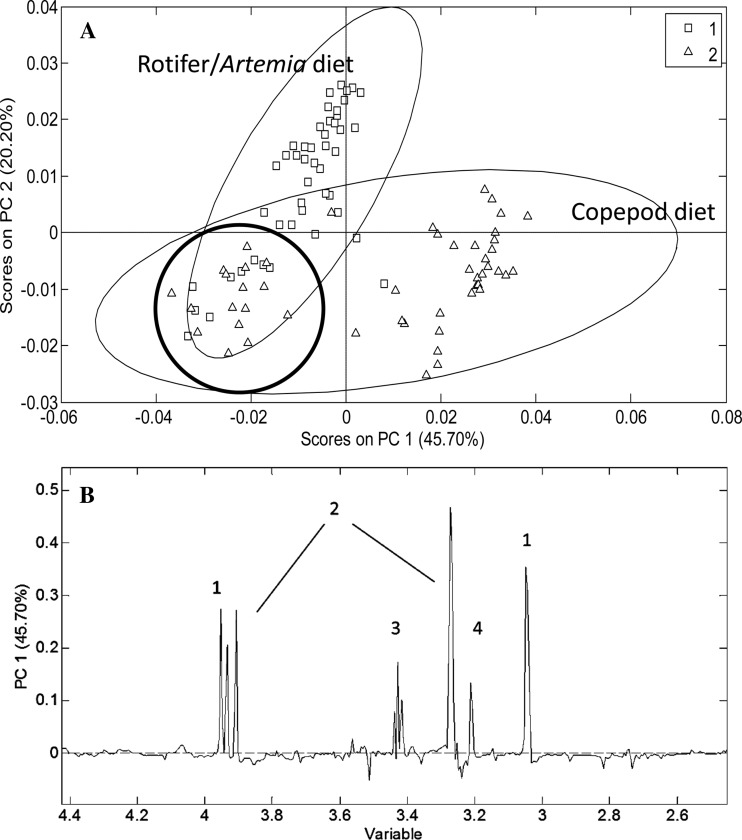
**Principal component analysis (PCA) of cod larvae from developmental stages 1-5 in the nutrition experiment.** (A) Symbols refer to the different nutrion regimes: Rotifer/Artemia diet (square) and Copepod diet (triangle). The variance in PC1 accounts for 45.70% of the variance between the earlier stages 1-2 (bold circle) and successively older larvae (stages 3-5) towards the right. Larvae from stages 3 to 5 are also separated along the PC2 (20.20%) and the two different groups correspond to larvae from the two different diets (rotifers/*Artemia* versus copepods). (B) Loading plot of the first principal component (PC1) in the PCA analysis. Numbered peaks show (1) creatine, (2) betaine/TMAO, (3) taurine, and (4) choline.

Larvae of different sizes/development stages were analyzed by NMR, and individuals up to stage 2 grouped together (Fig. 2A). Older individuals (stages 3-5), however, displayed clear separation between those fed rotifers/*Artemia* and those fed copepods. Also here the older larvae were analyzed individually and the score plot shows some inter-individual variance. The PC1 loading plot indicates that the larvae fed copepods have higher levels of creatine, betaine/TMAO, Tau and choline than those fed rotifers/*Artemia* (Fig. 2B). The PC2 loading plot (not shown) indicated a higher content of Ala and lactate, and less Tau in the larvae that were fed rotifers/*Artemia*.

## DISCUSSION

The metabolomic ‘snapshots’ of the cod larvae subjected to different temperatures and nutrient regimes show that the larvae displayed metabolomic differences during the various stages from hatching through first feeding to weaning onto a formulated feed. When discussing data based on NMR analysis, it is necessary to discern the ‘biological noise’ or the inherent metabolomic variability, from the true effects of perturbations or the experimental setup in the metabolomics data. Inter-individual differences can mask the experimental effects, and we therefore discuss our observations both as a function of developmental stage *and* potential effects of experimental setup (temperature and nutrition), and consider this approach important in order to understand the development of cod larvae between hatching and juveniles.

Our data showed little effect of the temperature treatments on growth in the early life of the larvae, and it was only after 20-25 dph (stage 2) that the growth of the T2 and T4 larvae began to increase rapidly. The larvae in T1 were exposed to low temperatures throughout development, thus the growth was slow throughout. In the PCA score plot larvae grouped into two clusters based on the metabolomic analysis (Fig. 1A). Younger larvae (until stage 2) showed a high score on PC1 (Fig. 1B) which is related to increased TMAO/betaine content and less creatine, Tau, Ala and lactate. In the older larvae (stages 3-5), there was a differentiation between the samples from the T2 and the T4 temperature regimes: The T2 larvae were subjected to low temperature during the egg phase, and they showed a higher score also on the PC2. This indicated that the betaine/TMAO, Ala and lactate levels were higher in the T2 larvae than in the T4 larvae, possibly reflecting the development of osmoregulation and muscle activity over the time of the experiment. The T1 larvae, which were exposed to low temperature from the egg stage also showed a lower score on the PC2, tentatively reflecting slower development of osmoregulation and muscles due to the low temperature.

The Tau content was more prominent in older larvae (stages 3-5), probably because the larvae were fed *Artemia* from stage 2 (25-46 dph), and *Artemia* contains more Tau than rotifers (Karlsen et al., 2015). The period between hatching and metamorphosis into juveniles involves many complex processes of functional organ development: e.g. the gastrointestinal tract, osmoregulatory systems, muscles and sensory organs, and ossification (Kjørsvik et al., 2004; Sæle, Ø., personal communication). The amino acid Tau has many important roles in metabolism (Chatzifotis et al., 2008; Salze and Davis, 2015), and functions as a buffering agent in mitochondria in animal cells (Hansen et al., 2010) and as a compatible solute, i.e. metabolites which interact without perturbing other molecules (Yancey, 2005). A study showed that Tau was higher in eggs from wild broodstock of cod compared to captive broodstock (Lanes et al., 2012). Hamre et al., showed that Tau was lower in cultured than in wild Ballan wrasse (*Labrus berggylta*) juveniles and mature gonads (Hamre et al., 2013a). This may indicate that Tau may be a limited nutrient in reared fish, and Tau requirement in marine fish must probably be assessed for each species and life-stage (Hawkyard et al., 2014).

The differences in metabolite concentrations between stage 3-5 larvae in the T2 and T4 groups was caused by different rearing temperatures in the embryonic stage. The effect lasted until the late larval stage even though the temperature conditions during larval rearing were similar between treatments. Embryonic temperature is known to affect muscle development and protein expression in fish larvae (Hall et al., 2003), and increased embryonic temperature shortens the embryonic phase and stimulates white muscle growth in cod larvae (Galloway et al., 1998, 1999).

In the nutrition experiment, clear effects of diet on larval size were observed after approximately 22 dph (stage 2), and the cod larvae that were fed copepods were bigger than the larvae that were fed rotifers/*Artemia* (Karlsen et al., 2015). Bigger, fast growing larvae are believed to be more robust, and a more nutritious diet is therefore considered as a paramount factor in cod rearing. The nutrition experiment showed a clear difference in the metabolome between the larvae that were fed different diets (Fig. 2A,B), and the larvae fed copepods had higher contents of creatine, betaine/TMAO and Tau. In addition choline was higher in larvae fed copepods. Analyses of the live feed showed that the principal differences between the rotifer/*Artemia* and the copepod diet was a higher content of protein (40 vs 60%) and Tau (1 vs 50 µmol/g DW) in copepods (Karlsen et al., 2015). Dietary differences may therefore be the reason that copepod-fed larvae seem to have a higher content of Tau and choline than larvae fed rotifers/*Artemia*. Similar differences were found for ballan wrasse larvae fed cultivated copepod nauplii or rotifers (Øie et al., 2015). There were also differences in the composition of fatty acids between rotifer/*Artemia* and copepod diets, including ARA, EPA and DHA and a minor difference in the ratio of polar to neutral lipids. With the NMR protocol applied here, however, large molecules like lipids and proteins are not observed and are therefore not represented in the data.

The higher creatine and TMAO/betaine in the copepod-fed larvae may be coupled to more muscle tissue and higher dietary concetrations of the methyl donors betaine and methionine, the latter due to a higher total protein content in the copepods. Choline and compounds containing choline, such as phosphatidylcholine, are the sources of TMAO and betaine (Seibel and Walsh, 2001). TMAO/betaine are important in osmoregulation, and efficient osmoregulation must be in place when the larvae hatch. TMAO also acts as a stabilizer of macromolecules, such as proteins, and protects them from unfolding and denaturing, for example in response to salt and heat (Yancey, 2005). Furthermore, betaine is one of several methyl-donors which re-activate methionine in the S-adenosylmethionine pathway (Michel et al., 2006). It is possible that rotifers and *Artemia* are deficient in choline, since they usually have low phospholipid contents (Hamre et al., 2013b).

Creatine stores energy in the muscle as creatine-phosphate, reversibly synthesized using a phosphate group from ATP. It is present in a much higher concentration than ATP and is necessary for prolonged muscle activity (Wallimann et al., 2011). Creatine is synthesized mainly in the liver and kidneys from arginine and glycine, using a methyl group from methionine (Cantoni and Vignos, 1954; Wallimann et al., 2011). Alanine and lactate are also related to muscle activity. The muscle uses amino acids as fuels, and the amino groups are shunted over to alanine while the carbon skeletons can be converted to lactate during anaerobic metabolism. Both compounds are transported to the liver for further metabolism (Felig, 1973). The increases of alanine and lactate in older larvae may therefore reflect increasing muscle mass and activity.

Metabolism is a complex and very dynamic system, and perhaps even more so in fish which undergo metamorphosis. Furthermore, a major determinant of how the digestive system (and indirectly the metabolome) functions is the microbiome (Llewellyn et al., 2014) that is established in the gastrointestinal (GI) system after the larvae open up their mouth and start feeding. Larvae from different experimental tanks do not necessarily host the same bacteria consortia (Bakke et al., 2013) and there is also a natural evolution in bacteria consortia during ontogeny (De Schryver and Vadstein, 2014). The microbial ecology of developing fish larvae should therefore be considered in future studies.

The findings presented here show some of the many important metabolic processes that must develop properly during the ontogeny of larvae: a robust osmoregulatory system including different osmolytes such as betaine/TMAO and Tau, a myriad of proteins built from the various amino acids we observed, muscle function (with functional compounds such as lactate) to catch prey and avoid predators, energy creation and consumption from ATP and creatine, to mention some. Temperature during the egg period, hatching and ontogeny is also a powerful modulating factor, and perhaps especially so during the very first stages until the larvae reach a certain size and robustness. Our data also show the importance of nutrient availability from the first feeding starts. The cod larvae are able to synthesize many essential metabolites, but at some stage the need for exogenously supplied metabolites is apparent. One example is the case with Tau seen here, which seems to be supplied to a large extent through the diet. On the other hand, temperature determines the speed and sometimes the order of development and the timing of exogeneous feeding, so temperature is a modulator of nutritional deficiency appearances. Further research is needed to understand and solve challenges related to high mortality of young larvae, poor physiological conditions, or malformations in the survivors. Data on the larval metabolome should be coupled to analyses of gene expression, to determine the properties which are regulated at the genetic level and post-translational modifications potentially related to temperature or nutrition.

## MATERIAL AND METHODS

The two experiments on effects of temperature and nutrition in cod larvae are described briefly below. A detailed description of the temperature experimental setup is presented elsewhere (Puvanendran et al., 2013), as well as the nutritional experimental setup (Karlsen et al., 2015; Penglase et al., 2015). For the temperature experiment, eggs from broodstock at Nofima in Tromsø were used, whereas eggs from broodstock at the Institute of Marine Research (Austevoll) were used for the nutrition experiment.

### Temperature experiment

The temperature experiment consisted of four different treatments, where the first one (T1) served as control: eggs and larvae were kept at low temperatures of 4.5±0.5°C, 5.5±0.5°C, and 7.5±0.5°C, respectively. In the second treatment (T2), eggs were kept at a low temperature (4.5±0.5°C) until hatching, and subjected to a high temperature (11.5±0.5°C) after hatching. In the third (T3) and forth (T4) treatments, eggs were subjected to a high temperature (9.5±0.5°C) until hatching and then split; the larvae were subjected to either a low (5.5±0.5°C; T3) or high (11.5±0.5°C; T4) temperature. Nannochloropsis (Instant Algae, Reed Mariculture Inc., CA, USA) was added to the tanks for the first ten days, and the larvae were fed rotifers enriched with Phosphonorse (Tromsø Fiskeindistri AS, Tromsø, Norway) and Micronorse (Tromsø Fiskeindistri AS, Tromsø, Norway) from 2-29 dph. From 25-46 dph the larvae were fed *Artemia*, enriched with Larviva Multigain (Biomar AS, Norway), Phosphonorse and Micronorce, and co-fed with a formulated feed from 38-44 dph. After a gradual decrease in *Artemia* feeding, the larvae were weaned onto a formulated feed (AlgoNorse Coldwater, Tromsø Fiskeindistri AS, Tromsø, Norway) between 45-56 dph.

### Nutrition experiment

Cod eggs were incubated at 5.8-6°C, using continuous light and water (35 ppt salinity) exchange. Post-hatch larvae (4 dph) were transferred to black start feeding tanks, and the temperature was successively increased from 8 to 11.6°C at 11 dph. Gentle flow and aeration was applied, and a 16:8 light:dark period. Prior to feeding the tanks were supplied with algae paste to produce green water conditions. From 4 dph, the cod larvae were fed a diet of either (i) enriched rotifers (*Brachionus* sp., 4-31 dph), followed by enriched rotifers and *Artemia* (32-35 dph) and only enriched *Artemia* from 36-63 dph, or (ii) harvested marine zooplankton with a high content of copepod, from 4 until 44 dph (matching the rotifer diet from 4-63 dph). Zooplankton was collected from a pond system (van der Meeren et al., 2014), and was provided in size fractions from nauplii (4-20 dph) to copepodites (from 20 dph) as the cod larvae increased in size. Larvae from both treatments were weaned onto formulated feed (AgloNorse 400-600 μm, Tromsø Fiskeindustri AS, Tromsø, Norway) when they reached 12-15 mm SL.

### Larval developmental stages

In the temperature experiment the larvae were sampled at standard lengths (SL, mm) corresponding to pre-, onset, mid- and end metamorphosis, respectively (Hunt von Herbing et al., 1996). In the nutrition experiment, on the other hand, sampling was performed at SLs corresponding to developmental stages 1-5 (Sæle, Ø., personal communication), and the corresponding ages (dph) therefore varied between treatments due to differences in growth rates. A comparison between the two experiments was made using fixed larvae (in 4% formaldehyde in phosphate buffered saline, pH 7.4, Apotekproduksjon AS; Norway) from both experiments. There were not a sufficient number of larvae to be sampled from the T3 treatment after day 29.

Bone staining with Alizarin Red (Kjørsvik et al., 2009) was done on 9-11 larvae from each treatment and each sampling time, and stained larvae were photographed while submerged in 40% glycerol, using a stereo microscope (Leica MZ7.5, Germany) equipped with a camera (Nikon Digital Sight DS-5M L1, Japan). Analyses of the larvae were performed both from the pictures and direct observations of the stained samples. SL was measured from the tip of the upper lip to the end of the vertebrae in preflexion larvae, and to the root of the caudal fin (peduncle) in postflexion larvae. Ossification of vertebrae and fin rays were included for a joint staging scale (Tables 2, 3).

### ^1^H NMR spectroscopy

#### Sample preparation

In both experiments cod larvae were sampled at selected stages and snap frozen in liquid nitrogen before freeze drying, transport, and storage at −80°C until the NMR analyses were performed. Larvae were extracted intact, and to adjust for the individual variation in body size from stages 1 to 4, 20 individuals were pooled in each sample. Individual larvae were analyzed from stage 5. Whole larvae were homogenized on a Precellys bead beater and extracted with 2:1 methanol:H_2_O. After centrifugation, 800 µl of the extract was transferred to a new tube and vacuum centrifuged for 30 min at 30°C to remove the MeOH. After quick freezing at −80°C, the extracts were lyophilized and stored cold and dark. Shortly before the NMR analysis, the lyophilized extracts were dissolved in 200 µl D_2_O/PBS buffer in 5 mm NMR tubes (Bruker), and 1 mM deuterated trimethylsilyl propanoic acid (TSP) was added for reference.

#### Data acquisition and processing

NMR spectroscopy was performed at the MR Core Facility, Norwegian University of Science and Technology (NTNU), and ^1^H NMR spectra were acquired on a 600 Mhz Bruker Avance III NMR spectrometer (Bruker Biospin GmbH, Rheinstetten, Germany) equipped with an autosampler (Sample Jet). Temperature during acquisition was 300 K and a 5 mm CPQCI cryoprobe was used to sample 1 D proton spectra with a preprogrammed water presaturation pulse sequence (*noesygppr1d,* Bruker library); the recycle delay was 3 s, and the mixing time 10 ms. Spectra were collected into 65 K data (SW 12,019 Hz) and the FID transformed with line broadening 0.3 Hz and zero filling 1.0. Phasing, baseline correction and chemical shift calibration (using the TSP signal as reference, δ 0.0 ppm) of the frequency domain spectra was done using Bruker TopSpin v. 3.0.

#### Spectral assignment and multivariate analysis

Chemical shifts were referenced to TSP as δ 0.0 ppm, and spectral assignment was performed using the metabolite library provided by Chenomx NMR suite v. 7.7. Processed spectra were analyzed using Matlab and the principal component analysis (PCA) routines included in PLS Toolbox (Eigenvector, v. 7.3.1). Spectral data between 10.0 and 0.2 ppm were binned into buckets of 0.05 ppm width, and the spectral region 4.92-4.42 (which contains the suppressed water signal) was removed. All spectra were normalized to unit area and mean-centered before they were included in the PCA model. The unsupervised PCA reduces a large dataset with many variable vectors to a low-dimensional, orthogonal projection were a few of the most significant variables are highlighted (the Principal Components). When these PCs have been identified, the loading plot of each PC was compared to the results of the spectral assignment to identify the metabolites that caused most of the variability in the spectra and identify and describe subgroups that display differences within the dataset.
